# Highly Efficient Regioselective Decanoylation of Hyperoside Using Nanobiocatalyst of Fe_3_O_4_@PDA-*Thermomyces lanuginosus* Lipase: Insights of Kinetics and Stability Evaluation

**DOI:** 10.3389/fbioe.2020.00485

**Published:** 2020-05-28

**Authors:** Yanhong Bi, Zhaoyu Wang, Yaoqi Tian, Haoran Fan, Shuo Huang, Yihui Lu, Zhengyu Jin

**Affiliations:** ^1^State Key Laboratory of Food Science and Technology, School of Food Science and Technology, Jiangnan University, Wuxi, China; ^2^School of Life Sciences and Food Engineering, Huaiyin Institute of Technology, Huai’an, China

**Keywords:** magnetic nanoparticle, hyperoside, immobilization, *Thermomyces lanuginosus* lipase, acylation

## Abstract

The immobilization of *Thermomyces lanuginosus* lipase on polydopamine-functionalized Fe_3_O_4_ magnetic nanoparticles (Fe_3_O_4_@PDA-TLL) as a nanobiocatalyst was successfully performed for the first time, and the Fe_3_O_4_@PDA-TLL was used for regioselective acylation of natural hyperoside with vinyl decanoate. The effects of several crucial factors, such as the reaction solvent, substrate molar ratio, temperature, and immobilized enzyme dosage, were investigated. Under optimum conditions, the reaction rate, 6″-regioselectivity, and maximum substrate conversion were as high as 12.6 mM/h, 100%, and 100%, respectively. An operational stability study demonstrated that the immobilized enzyme could maintain 90.1% of its initial maximum conversion even after reusing it five times. In addition, further investigations on the kinetic parameters, like *V*_max_, *K*_m_, *V*_max_/*K*_m_, and *E*_a_, also revealed that the biocompatible Fe_3_O_4_@PDA could act as an alternative carrier for the immobilization of different enzymes.

## Introduction

Hyperoside (also known as quercetin-3-*O*-galactoside or 3-*O*-β-D-galactopyranosyl quercetin), a type of flavonoid-*O*-glycoside, is the major pharmacological component of many traditional medicinal plants, such as *Hyperin perforatum* L., *Geranium carolinianum* L., *Zanthoxylum bungeanum*, *Crataegus pinnatifida* Bunge, and so forth (Pei et al., 2017). Extensive clinical studies have demonstrated that hyperoside exerts multiple bioactivities compared with those of quercetin, including anti-inflammatory, antidepressant, antitumor, and antihepatitis activities (Ku et al., 2015; Guan and Liu, 2016; Ahn and Lee, 2017; Guo et al., 2019).

Recently, natural product modification chemistry based on their privileged molecular skeletons has attracted increasing attention in the fields of biochemistry and pharmacology for the purpose of improving their biological activities and physicochemical properties (Guo, 2017; Li and Lou, 2018). As was shown in the review by Newman and Cragg (2016), 46% of the 1562 natural product agents approved over the past 34-year period are derived from their derivatives or analogs bearing natural compound pharmacophores. For example, flavonoid glycoside and their analogs usually exhibit the physicochemical properties of unsatisfactory lipid solubility, poor stability, and low bioavailability, owing to their exiting active natural polyphenol-rich structures, which limits their applications in lipophilic systems (Newman and Cragg, 2016; Yang et al., 2018). In order to circumvent these drawbacks, preparation of their ester derivatives has proved to be a promising strategy (Newman and Cragg, 2016; Rupasinghe, 2016). Warnakulasuriya and Sudan have reported that quercetin-3-*O*-glucoside derivatives with long aliphatic chains could significantly reduce the primary hepatocytes’ injury and improve inhibition of hepatocellular carcinoma cells compared to quercetin-3-*O*-glucoside itself (Sudan and Rupasinghe, 2015; Warnakulasuriya and Rupasinghe, 2016). Besides, recent experiments also provided evidence that ester derivatives of rutin (Xin et al., 2018), polydatin (Wang et al., 2017), anthocyanidin (Cruz et al., 2018), and naringin (de Araújo et al., 2017) exhibited enhanced biological activities, pharmacological activities, and structure stabilities compared to their corresponding parental compounds.

**SCHEME 1:**
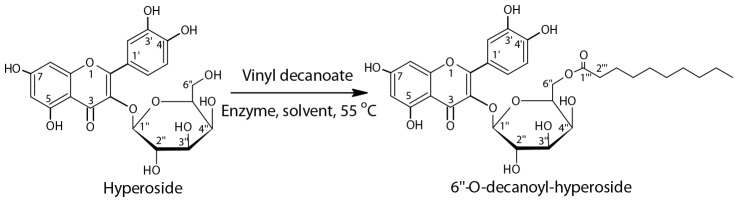
Fe_3_O_4_@PDA-TLL-catalyzed regioselective decanoylation of hyperoside.

Over the past few years, structural modifications by employing enzymatic methodology have clearly become an important topic in carbohydrate chemistry. This has been described as the preferred method, possessing a short synthetic route, impressive selectivity, and environmental friendliness compared to the multistep chemical approaches (de Araújo et al., 2017; Dunbar et al., 2017). However, it is embarrassing that the documented commercially available enzymes (e.g., Novozym 435, lipozyme TLIM, and PSIM), which act as the central role in catalytic processes, usually suffer from high cost and unsatisfactory organic solvent tolerance (Gonçalves Filho et al., 2019). Recently, magnetic Fe_3_O_4_ nanoparticles functioned with polydopamine (Fe_3_O_4_@PDA) have emerged as a desirable alternative to traditional materials for constructing immobilized enzymes (Liang et al., 2020). However, although several reports on Fe_3_O_4_@PDA-based immobilized enzymes, including cellulose, lipase, and ethanol dehydrogenase, have demonstrated the superiority of this method with a high ratio of enzyme to substrate, satisfactory enzyme stability, and facilitation of the separation and recovery for reuse (Chen et al., 2017; Liang et al., 2020), very few reports pay close attention to the enzymatic kinetic parameters, like *V*_max_, *K*_m_, *V*_max_/*K*_m_, to unravel the behavior of the nanobiocatalyst. As a result of this, in this study, for the first time, the immobilized Fe_3_O_4_@PDA-*Thermomyces lanuginosus* lipase (Fe_3_O_4_@PDA-TLL) was selected as the promising nanobiocatalyst to identify its kinetic behavior in non-aqueous enzymatic systems, in which the model reaction was the regioselective acylation of hyperoside with vinyl decanoate (Scheme 1).

## Materials and Methods

### Materials

*Thermomyces lanuginosus* lipase (TLL, 3921 U/g) was obtained from Novozymes Co., Ltd., China. Hyperoside (≥98%) was from Sigma-Aldrich. Dopamine hydrochloride, 2-methyl tetrahydrofuran (MeTHF), and *t*-amyl alcohol were provided by Aladdin. Vinyl decanoate (≥99%) was purchased from TCI. All other chemicals were obtained from commercial sources and were of analytical grade. All the used organic reagents were previously dried by 4 Å molecular sieves for 48 h.

### Preparation of Fe_3_O_4_@PDA-TLL

The magnetic Fe_3_O_4_ nanoparticles were prepared according to the conventional coprecipitation method described by Lou et al. (Cao et al., 2017). A certain amount of magnetic Fe_3_O_4_ nanoparticles was dispersed and ultrasonically treated for 10 min in deionized water. Then, the same molar quantity of dopamine hydrochloride was added into the above suspension. The pH of the mixture was adjusted to 8.5 by the addition of 1.5 mol/L NaOH solution. After stirring for 24 h at room temperature, the polydopamine-coated magnetic nanoparticle (Fe_3_O_4_@PDA) was formed and collected with an external magnet and washed three times with deionized water. For enzyme immobilization, 2.4 mL of TLL (260 mg/mL) solution and 0.4 g of Fe_3_O_4_@PDA were mixed and added into 12 mL of phosphate buffer (50 mmol/L, pH 8.5) at 25°C. After stirring at 200 rpm for 4.0 h, the immobilized TLL was separated and continuously washed until no protein was detected. The TLL-loaded Fe_3_O_4_@PDA was named Fe_3_O_4_@PDA-TLL.

### Assay of Fe_3_O_4_@PDA-TLL Activity

The activity of Fe_3_O_4_@PDA-TLL was determined using the *p*-nitrophenyl palmitate (*p*-NPP) method with slight modifications (Soni et al., 2018). An assay reaction mixture containing 0.1 g immobilized enzyme, 0.9 mL Tris-HCl buffer (50 mM, pH 8.0), and 0.1 mL *p-*NPP solution (a quantity of 30 mg *p-*NPP was dissolved in 10 mL isopropanol) was incubated at 37°C for 10 min. After this, 5.0 mL 95% ethyl alcohol was added to inactivate the enzyme and measure the absorption at 410 nm. One unit of activity (U) was defined as the amount of enzyme required to produce 1.0 μmol *p*-nitrophenol (*p*-NP) in 1.0 min under the above conditions. The specific activity of the Fe_3_O_4_@PDA-TLL was 8022 U/g.

### Enzymatic Synthesis of Hyperoside Ester Derivative

In a typical experiment, 3.0 mL solvent containing 0.03 mmol hyperoside, a certain amount of Fe_3_O_4_@PDA-TLL and vinyl decanoate was incubated in a 10 mL Erlenmeyer shaking flask in a rotary shaker. Rotate speed was set at 200 rpm and the operating temperature was set as desired. Then, 20 μL of the reaction mixture was withdrawn at specified time intervals and diluted 50-fold with mobile phase. The mixture was centrifuged at 10,000 rpm for 5.0 min and the upper layer was drawn for HPLC analysis.

### Operational Stability of Fe_3_O_4_@PDA-TLL

The operational stability of Fe_3_O_4_@PDA-TLL was determined using recycling reactions. When the maximum hyperoside conversion was achieved in each reaction, the suspension was centrifuged and the supernatant was decanted. The reused enzyme was washed three times with the fresh reaction solvent and added into 3.0 mL MeTHF containing 0.03 mmol hyperoside and 0.33 mmol vinyl decanoate at 55°C and 200 rpm. Then, enzyme residual activity and maximum substrate conversion were measured. The initial activity and maximum conversion received in the first batch were defined as 100%.

### Determination of Kinetic Constants and Apparent Activation Energy (*E*_a_)

The concentrations of the hyperoside used to determine the kinetic constants of enzymatic acylation in different solvents were THF (2.0–20 mM), MeTHF (2.0–20 mM), dioxane (5.0–35 mM), and *t*-butanol (2.0–20 mM). All experiments were conducted under the optimal reaction conditions obtained by a single-factor experiment. The kinetic constants (*K*_m_ and *V*_max_) were calculated from Hanes-Woolf plots. *E*_a_ was calculated according to the linear regression analysis of the Arrhenius plot.

### HPLC Analysis and Structure Determination of the Esters

The reaction mixture was analyzed by HPLC on a 4.6 mm × 250 mm (5 μm) Zorbax XDB-C18 column (Agilent Technologies Industries Co., Ltd., United States) using an Agilent G1311A pump and a UV detector. The mobile phase was a mixture of methanol and water (80/20, v/v) with a flow rate of 1.0 mL/min. The UV absorption wavelength for HPLC analysis and retention times for hyperoside and 6″-*O*-decanoyl hyperoside were 360 nm, 2.50 min, and 6.11 min, respectively. All reported data were averages of experiments performed at least in duplicate. The product was purified by silica gel chromatography with an eluent consisting of petroleum ether/ethyl acetate/methanol (5/10/2, v/v/v). Structural assignments were made on the basis of the changes in the ^13^C NMR (100 MHz) and ^1^H NMR (400 MHz) spectra caused by the acylation (Bruker DRX-400 NMR Spectrometer, Bruker Co., Germany). Results from the NMR spectroscopy were recorded as follows: ^1^H NMR (DMSO-*d*_6_) δ: 12.63 (1H, s, 5-OH), 7.64 (1H, dd, *J* = 8.5, 2.2 Hz, H-6′), 7.49 (1H, d, *J* = 2.2 Hz, H-2′), 6.81 (1H, d, *J* = 8.5 Hz, H-5′), 6.39 (1H, d, *J* = 2.0 Hz, H-8), 6.18 (1H, d, *J* = 2.0 Hz, H-6), 5.38 (1H, d, *J* = 7.8 Hz, H-1″), 5.21 (1H, br s, -OH), 4.95 (1H, br s, -OH), 4.69 (1H, br s, -OH), 4.11 (1H, dd, *J* = 11.4, 8.4 Hz, H-5″), 3.91 (1H, dd, *J* = 11.4, 3.8 Hz, H-6″a), 3.61 (1H, m, H-4″), 3.50–3.40 (3H, m, H-3″, 6″b, H-2″), 2.00–1.93 (2H, m, H-2″′), 1.24–1.01 (14H, m, H-3″′-9″′), 0.86 (3H, t, *J* = 7.1 Hz, H-10″′). ^13^C NMR (DMSO-*d*_6_) δ: 177.9 (C-4), 172.9 (C-1″′), 164.6 (C-7), 161.7 (C-5), 156.6 (C-2), 153.6 (C-9), 148.9 (C-4′), 145.3 (C-3′), 133.7 (C-3), 122.4 (C-6′), 121.5 (C-1′), 116.2 (C-2′), 115.6 (C-5′), 104.2 (C-10), 101.8 (C-1″), 99.1 (C-6), 93.9 (C-8), 75.0 (C-5″), 73.4 (C-3″), 71.4 (C-2″), 68.8 (C-4″), 63.6 (C-6″), 33.7 (C-2″′), 31.8 (C-8″′), 29.3–28.8 (C-4″′-7″′), 24.7 (C-3″′), 22.6 (C-9″′), 14.4 (C-10″′).

## Results and Discussion

### Effect of the Reaction Medium

In non-aqueous biotransformation reactions, the reaction medium plays a determinant role and modulates the enzyme properties, like enzyme activity, selectivity, and stability (Elgharbawy et al., 2018). To date, no empirical rules could be used for reference to guide the use of media during enzymatic synthetic processes; trial and error procedures were still used as an essential method for solvent choice. In this content, the acylation of hyperoside was performed in 11 organic solvents with different natures, as listed in Table 1.

**TABLE 1:** Effect of medium on Fe_3_O_4_@PDA-TLL-catalyzed decanoylation of hyperoside.

Medium	Log *P*	Viscosity^*a*^	*V*_0_ (mM/h)	Time (h)	*C*^*b*^ (%)	6″-Regioselectivity^*c*^ (%)
DMSO	–1.30	2.24	–	3.0	2.7 ± 0.1	100
Dioxane	–1.10	1.30	3.8 ± 0.1	14.0	43.7 ± 0.5	100
DMF	–1.00	0.92	–	3.0	3.5 ± 0.1	100
Acetonitrile	–0.33	0.37	5.0 ± 0.2	14.0	65.6 ± 1.0	100
Acetone	–0.23	0.32	5.7 ± 0.2	14.0	63.3 ± 0.9	100
THF	0.49	0.55	6.0 ± 0.3	12.0	70.0 ± 1.5	100
*t*-Butanol	0.60	3.30	2.8 ± 0.1	16.0	50.7 ± 0.5	100
Pyridine	0.71	0.97	–	4.0	6.7 ± 0.1	100
MeTHF	0.99	0.60	7.1 ± 0.3	12.0	78.5 ± 1.2	100
*t*-Amyl alcohol	1.15	3.70	5.1 ± 0.2	14.0	59.6 ± 0.7	100
Cyclohexanone	1.43	2.20	2.7 ± 0.1	14.0	47.5 ± 1.3	100

*Reaction conditions: 0.03 mmol hyperoside, 0.21 mmol vinyl decanoate, 180 mg Fe_3_O_4_@PDA-TLL, 3.0 mL anhydrous solvent, 45°C, 200 rpm. ^*a*^The viscosity of medium at 20°C. ^*b*^Maximum conversion. ^*c*^Regioselectivity was defined as the ratio of the concentration of the indicated product to that of all the products formed.*

It can be clearly seen that the solvent polarity affected the reaction rate and substrate conversion more dramatically than the regioselectivity. However, the catalytic performance of Fe_3_O_4_@PDA-TLL could not be correlated well with log *P*-values ranging from −1.30 to 1.43 of the organic solvent, which is in agreement with our previous reports (Wang Z. Y. et al., 2015; Wang et al., 2016). Fortunately, the immobilized Fe_3_O_4_@PDA-TLL evidenced the moderate to good catalytic behavior in most of the tested solvents, and the highest reaction rate and conversion were found in the eco-friendly MeTHF with 7.1 mM/h and 78.5%, respectively. DMSO, DMF, and pyridine severely deactivated the immobilized enzyme activities. Except for the well-known factor of solvent polarity affecting the hydration water of the enzyme molecule, the solvent penetration ability into the enzyme active site, enzyme protein conformation change, and solubility of the substrate and product unavoidably influenced the enzymatic processes (Yang et al., 2004; Sanchez-Fernandez et al., 2017). With regarding to the regioselectivity, it was interesting to find that Fe_3_O_4_@PDA-TLL showed favor toward the 6″-OH of hyperoside in all the media assayed. The most possible reason might be that the polyphenol hydroxyl structure of the hyperoside afforded the specific substrate-binding pattern in the catalytic pocket of the enzyme active site. Therefore, the more active and less-hindered 6″-primary hydroxyl may enter into the active site of the enzyme more easily than other hydroxyls to attack the acyl-enzyme transition-state intermediate and form the 6″-*O*-monoester derivative.

### Optimization of Fe_3_O_4_@PDA-TLL Production of Decanoyl Hyperoside

**FIGURE 1:**
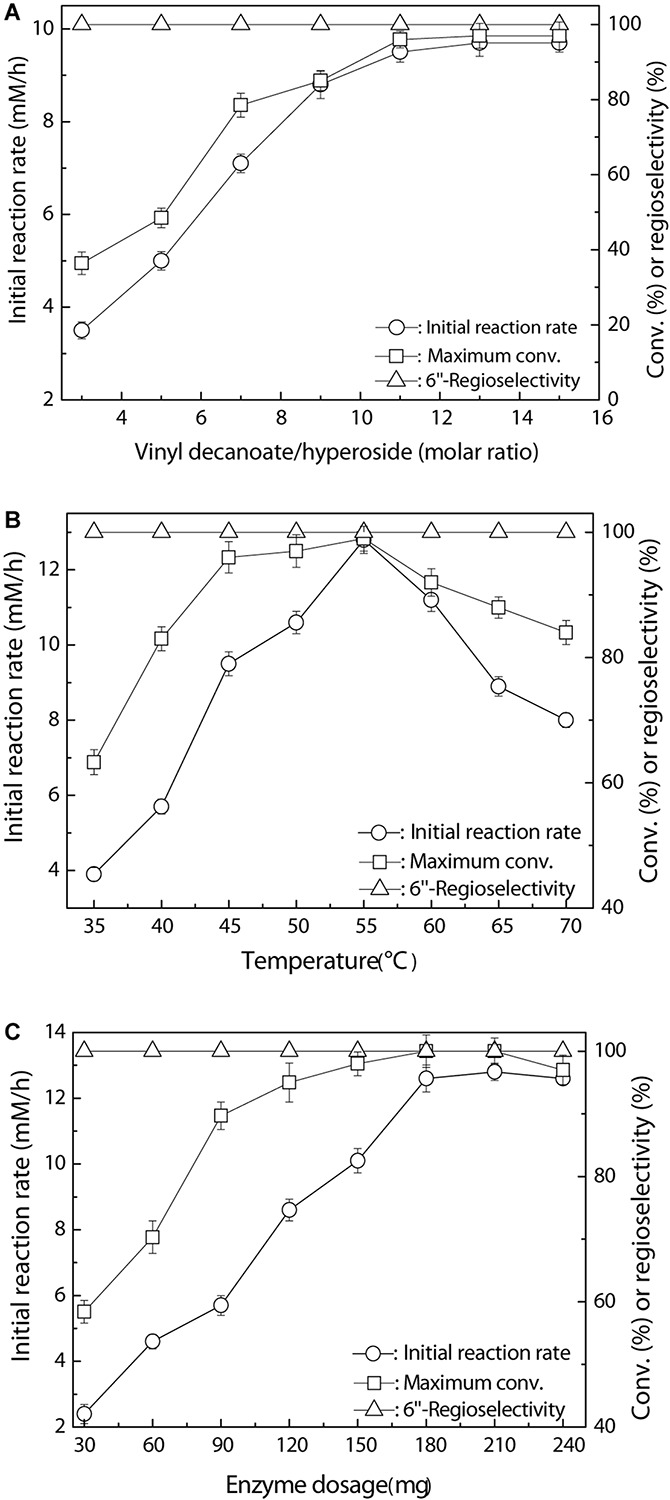
Regioselective decanoylation of hyperoside catalyzed by Fe_3_O_4_@PDA-TLL. **(A)** Effect of the molar ratio of vinyl decanoate to hyproside (0.03 mmol hyproside, 3.0 mL anhydrous MeTHF, 180 mg enzyme, various amounts of vinyl decanoate at 45°C, 200 rpm). **(B)** Effect of the temperature (0.03 mmol hyperoside, 0.33 mmol vinyl decanoate, 3.0 mL anhydrous MeTHF, 180 mg Fe_3_O_4_@PDA-TLL at different temperatures, 200 rpm). **(C)** Effect of the enzyme dosage (0.03 mmol hyperoside, 0.33 mmol vinyl decanoate, 3.0 mL anhydrous MeTHF, various amounts of the Fe_3_O_4_@PDA-TLL at 55°C, 200 rpm).

To further understand and improve the catalytic performance of the new immobilized Fe_3_O_4_@PDA-TLL, several key variables, such as the substrate molar ratio, reaction temperature, and enzyme dosage in the reaction, were examined in detail. In general, an excessive amount of acyl donors is normally required, owing to the presence of side reactions of enzymatic hydrolysis of vinyl ester and acylated product (Amanda Gomes Almeida et al., 2017). As depicted in Figure 1A, the substrate molar ratio displayed a great effect on the behavior of the immobilized biocatalyst. A striking improvement in initial reaction rate and substrate conversion was observed with increasing the molar ratio of vinyl decanoate to hyperoside up to 11, which is the optimal ratio of the double substrates. Figure 1B shows the enzymatic decanoylation of hyperoside affected by the temperature, with 55°C being optimal; a high temperature may induce a significant conformational unfolding of the enzyme, resulting in decreasing the initial rate and conversion. Moreover, the optimum enzyme dosage was 180 mg with the excellent conversion of 99.0%, and no substantial variation in acylation rate occurred while further increasing the enzyme dosage up to 240 mg (Figure 1C). Additionally, it is worth emphasizing that change in the TLL immobilized carrier of Fe_3_O_4_@PDA had a marginal effect on the regioselectivity among the examined key reaction conditions. Very similar results concerning the primary hydroxyl regioselectivity were also obtained by Ghasemi and co-workers in the regioselective acetylation of prednisolone using TLL lipase glutaraldehyde-mediated immobilization on Fe_3_O_4_ nanoparticles (Ghasemi et al., 2013).

### Time Course of Enzymatic Reaction and Operational Stability

**FIGURE 2:**
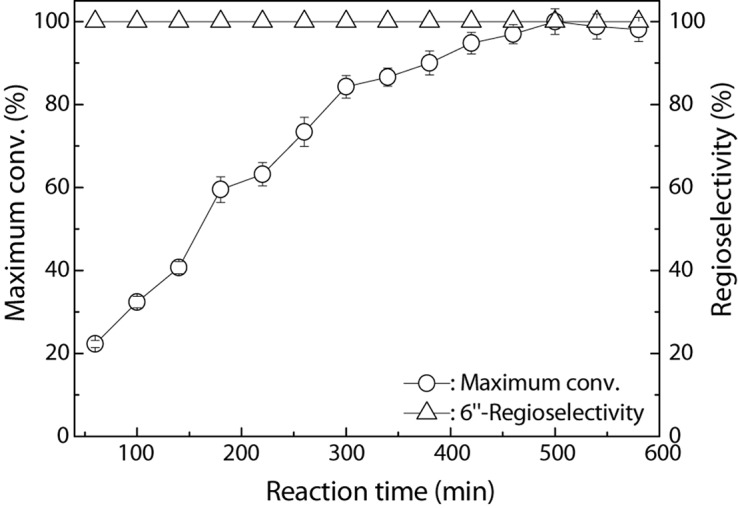
Process curve of Fe_3_O_4_@PDA-TLL-catalyzed decanoylation of hyperoside. Reaction conditions: 0.03 mmol hyperoside, 0.33 mmol vinyl decanoate, 3.0 mL anhydrous MeTHF, 180 mg Fe_3_O_4_@PDA-TLL, 55°C, 200 rpm.

The time course of the Fe_3_O_4_@PDA-TLL-mediated preparation of the 6″-*O*-decanoyl derivative of hyperoside is shown in Figure 2. The hyperoside conversion went up rapidly within 300 min and then there was a smooth rise, possibly due to the lower concentration of the double substrates. During the enzymatic acylation process, 6″-*O*-decanoyl hyperoside was the end product, with a regioselectivity of 100%.

**FIGURE 3:**
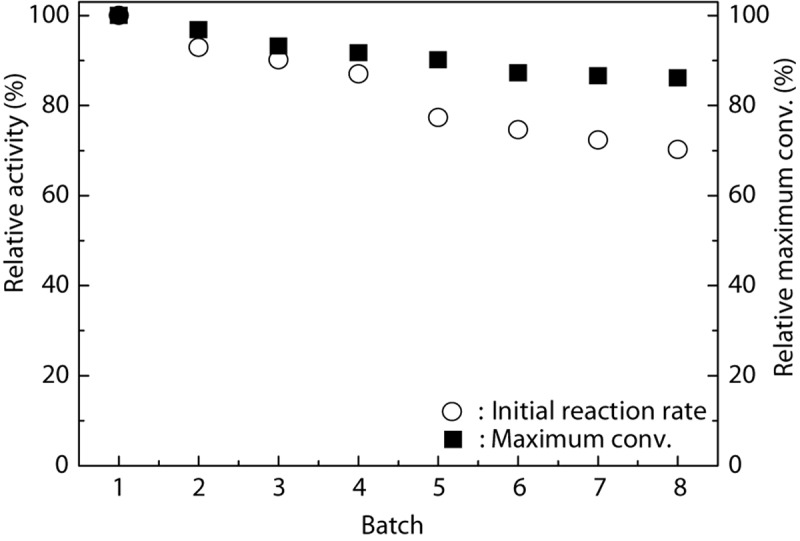
Operational stability of Fe_3_O_4_@PDA-TLL in MeTHF. Reaction conditions: 0.03 mmol hyperoside, 0.33 mmol vinyl decanoate, 3.0 mL anhydrous MeTHF, 180 mg Fe_3_O_4_@PDA-TLL, 55°C, 200 rpm.

From an industrial point of view, good operational stability and reusability of the catalyst are necessary for fine chemical production. Wang X. Y. et al. (2015) successfully immobilized TLL onto the Fe_3_O_4_@chitosan nanoparticles and checked their reusability. The results revealed that the Fe_3_O_4_@chitosan-TLL showed a preferable stability and retained 70% of its initial activity after ten reuses. Figure 3 shows that the Fe_3_O_4_@PDA-TLL exhibited satisfactory stability. Although the enzyme kept 77.3% of its original activity in MeTHF after being reused for five batches, the relative maximum conversion still remained about 90.1%, suggesting that this immobilized enzyme displayed good organic solvent tolerance and that the immobilized carrier can greatly improve the reuse times of the enzyme as well as the efficiency of the process.

### Determination of Kinetic Constants and Apparent Activation Energy (*E*_a_)

**FIGURE 4:**
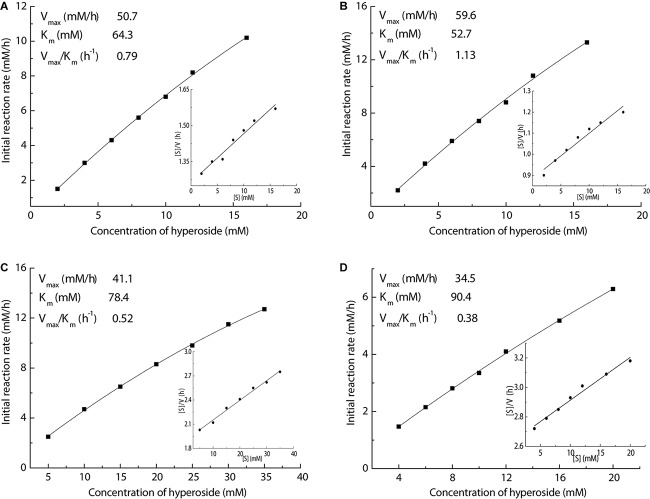
Effect of the hyperoside concentration on enzymatic acylation in various media (inset of Hanes–Woolf plot). Reaction conditions: different concentrations of hyperoside, vinyl decanoate (11 equiv.), 3.0 mL anhydrous solvent [**(A)** THF, **(B)** MeTHF, **(C)** dioxane, **(D)** *t*-butanol], 180 mg Fe_3_O_4_@PDA-TLL, 55°C, 200 rpm.

In order to gain an in-depth insight into the superiority of this Fe_3_O_4_@PDA-TLL, organic solvents containing THF, MeTHF, dioxane, and *t*-butanol were selected to measure the kinetic parameters, including *K*_m_, *V*_max_, and *V*_max_/*K*_m_, by using the linear form of Hanes-Woolf plots. As illustrated in Figure 4, the new immobilized TLL exerted the highest affinity in MeTHF for the substrates, which was evidenced with the highest *V*_max_ (59.6 mM/h) and lowest apparent *K*_m_ (52.7 mM) values in the above enzymatic acylation systems containing the tested solvents. Excellent catalytic efficiency with the highest *V*_max_/*K*_m_ of 1.13/h also demonstrated that MeTHF was the most effective medium in this enzymatic reaction.

**FIGURE 5:**
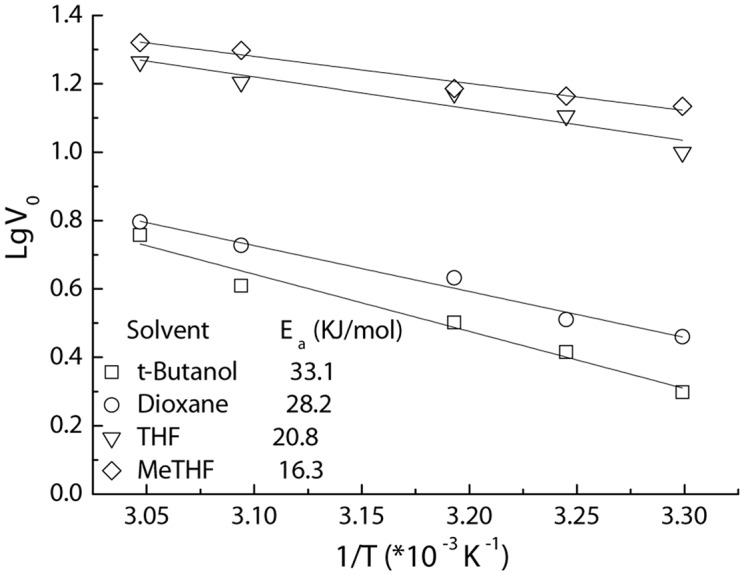
Arrhenius plots of Fe_3_O_4_@PDA-TLL-catalyzed acylation of hyperoside in various media. Reaction conditions: 0.03 mmol hyperoside, 0.33 mmol vinyl decanoate, 3.0 mL anhydrous solvent, 180 mg Fe_3_O_4_@PDA-TLL, different temperatures (in the range of 35–55°C), 200 rpm.

Furthermore, the apparent activation energy (*E*_a_) for the acylation is also assayed using Arrhenius plots (Figure 5). The *E*_a_ value of 16.3 KJ/mol for the MeTHF system afforded by Fe_3_O_4_@PDA-TLL was much lower than those received in other media (20.8–33.1 KJ/mol), indicating that MeTHF is beneficial to accelerate the enzymatic reaction and enhance this immobilized enzyme’s stability. These kinetic studies on the reactions described above are very similar to the TL IM (*T. lanuginosus* lipase immobilized on granulated silica)-mediated acylations (Wang et al., 2016), which suggests that the replacement of the immobilized carrier still retained the excellent characteristics of the enzyme itself and that Fe_3_O_4_@PDA could act as an alternative carrier for enzyme immobilization.

## Conclusion

In conclusion, *T. lanuginosus* lipase was successfully immobilized onto the biocompatible nanoparticles of Fe_3_O_4_@PDA and showed satisfactory performance, with absolute 6″-position, higher initial rate, and substrate conversion during the synthesis of the decanoyl derivative of hyperoside. Detailed investigations on the operational stability and kinetic studies also addressed that the immobilization of enzymes, using this method, could be a good and practical option for various industries. These findings will undoubtedly enrich the application of the novel immobilized carrier in the biotransformation fields.

## Data Availability Statement

All datasets generated for this study are included in the article.

## Author Contributions

ZW and ZJ gave the idea for this project and helped in the case of scientific problems. YB was responsible for planning and performing the experiments as well as writing the main part of the manuscript. SH and YL helped with the experiments operation and data collation. YT and HF were responsible for the final correction and English proofing.

## Conflict of Interest

The authors declare that the research was conducted in the absence of any commercial or financial relationships that could be construed as a potential conflict of interest.
